# La différence épidémiologique des hémorragies digestives hautes entre les hommes et les femmes

**Published:** 2012-08-02

**Authors:** Amine El Mekkaoui, Kaoutar Saâda, Ihssane Mellouki, Mounia El Yousfi, Nourdin Aqodad, Mohammed El Abkari, Adil Ibrahimi, Dafr-Allah Benajah

**Affiliations:** 1 Service d'Hépato-gastro-entérologie, CHU Hassan II - Faculté de Médecine et de Pharmacie de Fès - Université Sidi Mohammed Ben Abdellah de Fès, Maroc

**Keywords:** Hémorragie digestive haute, épidémiologie, sexe masculin, sexe féminin, endoscopie, ulcère gastroduodénal, hypertension portale, upper gastrointestinal bleeding, epidemiology, male, female, endoscopy, gastroduodenal ulcer, portal hypertension

## Abstract

**Introduction:**

Des différences épidémiologiques, étiologique voire pronostique des hémorragies digestives hautes (HDH) entre les deux sexes opposés ont été cité par différentes études.

**Méthodes:**

Nous avons essayé de déceler ces différences à travers une analyse rétrospective nichée sur une étude prospective sur les hémorragies digestives hautes ayant inclus 945 patients.

**Résultats:**

Six cents trente-sept patients étaient des hommes (67,4% Vs 32,6%). Un antécédent d'HDH était trouvé chez 24,2% des cas sans différence significative entre les deux sexes. L'âge de survenue de l'hémorragie était plus élevé chez les femmes que chez les hommes : 51,5 ans ± 18,8 Vs 47,8 ans ± 18,3 (p : 0,003). Les étiologies de l'HDH étaient différentes entre les deux sexes. Alors que l'hémorragie liée à l'HTP était la première cause chez la femme (38 % Vs 23,5 % chez l'homme, p<0,0001), c'est la pathologie ulcéreuse qui venait en premier chez l'homme (62 % Vs 36,7 % chez la femme, p<0,0001). Un besoin transfusionnel était noté chez 42,4 % des patients de sexe masculin contre 35,4 % des patientes avec un p = 0,03. Le taux de récidive et de décès global étaient de 7,5 % et de 5,7 % des cas respectivement, sans différence significative entre les deux sexes.

**Conclusion:**

L'étude trouve un profil épidémiologique, clinique et étiologique différent selon le sexe des patients.

## Introduction

Des différences de fréquence, d'âge, d'étiologie voire même de pronostic des hémorragies digestives hautes (HDH), entre les hommes et les femmes, ont été citées dans différentes études. Ces différents paramètres sont très hétérogènes et différemment appréciés d'une étude à une autre, d'autant plus que peu de publications ont cherché les particularités des HDH selon le sexe. Dans ce travail, nous avons essayé de déceler les différences démographiques, cliniques, étiologiques et évolutives des HDH liées au genre.

## Méthodes

Il s'agit d'une une étude prospective sur les hémorragies digestives hautes, sur laquelle nous avons réalisé une analyse rétrospective, comparant les différents paramètres entre les deux sexes opposés. Cette étude avait inclus tous les patients adultes âgés de plus de 16 ans, admis entre janvier 2005 et mai 2011, et ayant présenté une hématémèse et/ou un méléna objectivé cliniquement et/ou une anémie d'installation aigue, rattachée à une lésion du tube digestif haut et ayant bénéficié d'une fibroscopie œso-gastroduodénale.

La cause de l'HDH a été attribuée à une lésion donnée en présence d'une hémorragie active, de stigmates da saignement récent ou en l'absence d'autres causes de saignement. En cas de présence de plus d'une lésion susceptible de saigner, les deux ont été considérées comme étiologiques. Le délai de réalisation d'endoscopie reflétait le temps écoulé entre le premier épisode hémorragique et la réalisation de l'endoscopie.

La récidive hémorragique précoce a été définie par une récidive survenant dans les 5 jours suivant la réalisation de l'endoscopie soit d'une instabilité hémodynamique, d'une récidive d'hématémèse, d'une apparition de rectorragies, d'une persistance de méléna après trois jours ou une apparition de nouveau méléna après une coloration normale des selles ou d'une chute du taux d'hémoglobine de plus de 2g/dl dans l'espace de 24 heures. Le taux de mortalité était défini par la mortalité intra-hospitalière survenue au décours d'une HDH.

Les données épidémiologiques des malades, les renseignements cliniques, les résultats de l'endoscopie, les traitements reçus et l'évolution des patients au cours de leur hospitalisation ont été recueillis puis analysés par le logiciel EpiInfo 3.5.1. Le test de Student a été utilisé pour l'analyse des variables qualitatives, quant aux variables quantitatives, on a utilisé les tests de *X*<^2^ (Chi-2), de Fisher exact et le corrigé de Yates, selon leurs valeurs théoriques.

## Résultats

Durant la période d'étude nous avons inclus 945 patients, 637 étaient des hommes (67,4 %) et 308 des femmes (32,6 %). Chez les patients âgés d'au moins 65 ans, les hommes représentaient 58,1 % des cas. Au total, l'âge moyen était de 47,8 ± 18,3 ans de chez les patients de sexe masculin et de 51,5 ± 18,8 ans chez les patientes (p = 0,003). Un antécédent d'ulcère gastroduodénal et d'HDH étaient retrouvés chez 10 % versus 4,5 % (p = 0,003) et 24,3 % contre 24 % (p = 0,9) respectivement chez les hommes et les femmes (Tableau 1). Par ailleurs, une prise de médicaments gastro-toxiques était notée chez 25,9 % sans différence significative entre les deux sexes. Une hématémèse était présente dans 79,6 % des cas chez le sexe masculin contre 85,1 % chez le sexe opposé (p = 0,04) (Tableau 1) ; par contre un état de choc initial a été noté chez 2,8 des cas sans différence significative lié au genre. La moyenne des chiffres tensionnels artériels systoliques (TAS) et diastoliques (TAD) étaient de 11,2 cmhg ± 1,8 et de 6,6 cmhg ± 1 respectivement. La fréquence cardiaque moyenne était de 87,6 ± 11,8 battement par minute. Les patientes de sexe féminin avaient plus de thrombopénie et de taux de prothrombine (TP) inférieur à 70 % que chez les hommes mais la différence n'était significative que pour le TP bas, p = 0,4 et 6.10^-4^ respectivement.

**Tableau 1:** Caractéristiques démographiques, cliniques et para-cliniques des patients selon le genre

	Homme	Femme	Signification statistique (p)
**Effectif**	637	308	-
**Pourcentage**	67,4	32,6	-
**Âge**			
Moyenne	47,8 ± 18,3	51,5 ± 18,8	*3.10* ^*-3*^
Médiane [extrémités]	48 (16-110)	54 [16-93]	
≥ 65 ans	20,9	31,3	*5.10* ^*-4*^
**Antécédent**			
Médicament gastrotoxique	27,2	23,4	NS
Douleurs épigastriques	20,9	14,3	*1.10* ^*-2*^
Ulcère gastroduodénale	10	4,5	*3.10* ^*-3*^
Hémorragie digestive haute	24,3	24	NS
Comorbidités	9,3	10,7	NS
**Caractéristiques cliniques et paracliniques**			
Présence d'hématémèse	79,6	85,1	*4.10* ^*-2*^
Présence de méléna	78,8	62,3	*1.10* ^*-6*^
Méléna exclusive	18,8	14	NS
Réctorragies	2	2,6	NS
Etat de choc	2,7	2,9	NS
Syndrome anémique	55,4	51	NS
Douleur épigastrique	36,7	25,3	*4.10* ^*-4*^
Signes d'HTP	12,7	24,7	*4.10* ^*-6*^
TAS (cmhg)	11,3	10,9	NS
TAD (cmhg)	6,5 ± 0,9	6,6 ± 1	NS
Pouls (battement par minute)	87,3 ± 11,7	88,2 ± 12,1	NS
Hémoglobine (g/dl)	8,3 ± 3,2	8,4 ± 2,8	NS
Taux de plaquette (103/mm3)	195 ± 116	196 ± 109	NS
Thrombopénie	32,2	38,3	NS
Taux de prothrombine (TP en %)	84 ± 19,7	77,6 ± 22,9	*1.10* ^*-3*^
TP <70 %	23,1	36,3	*6.10* ^*-4*^

Les variables qualitatives sont exprimées par leur fréquence en pourcentage (%) et les variables quantitatives sont exprimées par leur moyenne ± écart-type. NS : non significatif. Les différences significatives (< 0,05) sont *en italique*.

Le délai médian de réalisation de l'endoscopie par rapport au premier épisode hémorragique était de 20 heures (1-360) chez les hommes et de 16 heures chez les femmes (1-360) (p = 0,18) sachant que 67,3 % des endoscopies étaient réalisées dans les 24 heures suivant l'épisode hémorragique ; chez les patients ayant exprimé leur hémorragie exclusivement par un méléna, ce délai était plus important avec une médiane de 24 heures (2-360) et de 48 heures (1-360) respectivement (p = 0,02). L'exploration endoscopique était jugée pathologique, non concluante, et normale chez 97,2 % versus 93,2 % (p = 3.10^-3^), 1,4 % versus 1,6 % (p = 0,5) et 1,4 % versus 5,2 % (p = 6.10^-4^) respectivement chez les hommes et les femmes.

Chez les hommes, les étiologies retrouvées étaient la pathologie ulcéreuse gastroduodénale (UGD) dans 62,0 % des cas, l'hémorragie liée à l'hypertension portale (HTP) dans 23,5 % des cas, une œsophagite et une gastro-duodénite aigue dans 11,5 % et 8,8 % des cas respectivement. Par contre chez les femmes, l'UGD représentait 36,7 % des cas (p < 1.10^-10^), l'hémorragie lié à l'HTP représentait la première cause par 38 % des cas (p = 4.10^-6^) et l'œsophagite ainsi que la gastro-duodénite représentaient respectivement 9,4 % (p = 0,3) et 16,6 % (p = 4.10^-4^) des cas.

Concernant la pathologie ulcéreuse, l'ulcère bulbaire représentait 41,8 % des cas d'HDH, dont 80,3 % des cas étaient des hommes, L'ulcère était classé stade I et II dans 7,7 % et 19,9 % respectivement. Par contre, l'ulcère gastrique ne représentait que 15,2 % des étiologies dont 71,5 % des cas étaient de sexe masculin (Tableau 2). Par ailleurs, une hémostase endoscopique était effectuée chez 6,3 % des ulcères gastroduodénaux et un seul patient avait nécessité une hémostase chirurgicale sans différence entre les deux groupes. Pour l'hémorragie liée à l'HTP, une rupture de varice œsophagienne était la cause dominante représentant 22 % des cas d'HDH, cette fréquence était répartie différemment entre les deux sexes (Tableau 2). Un traitement par drogue vaso-active était effectué chez 7 % des cas et une hémostase endoscopique était nécessaire dans 75,3 % des cas sans différence entre les deux groupes.

**Tableau 2:** Répartition des étiologies selon le genre et l'âge

	Homme			Femme			Signification statistique		
		< 65 ans	≥ 65 ans		< 65 ans	≥ 65 ans	p	p^a^	p^b^
Effectif (n)	637	504	133	308	212	96			
Pourcentage	67,4	53,3	14,1	32,6	22,4	10,2			
Ulcère bulbaire	49,8	54,6	32,3	25,3	24,2	28,1	*<1.10* ^*-10*^	*<1.10* ^*-10*^	NS
Forrest I	7,9	8,4	4,8	6,6	3,9	12,0	NS	NS	NS
Siège postérieur	27,0	26,0	33,3	30,3	29,4	32,0	NS	NS	NS
Ulcère gastrique	16,2	13,7	25,6	13,3	11,4	17,7	NS	NS	NS
Petite courbure	11,7	11,6	11,8	12,2	16,7	5,9	NS	NS	NS
Angulus	24,3	26,1	20,6	22,0	25,0	17,6	NS	NS	NS
Rupture de varice œsophagienne	17,9	16,5	23,3	30,5	34,4	21,9	*1.10* ^*-5*^	*1.10* ^*-6*^	NS
Gastrite érosive ou ulcérative	8,8	8,4	9,8	16,6	18,0	13,5	*4.10* ^*-4*^	*1.10* ^*-3*^	NS
Œsophagite	11,5	9,0	19,5	9,4	8,1	12,5	NS	NS	0,15
Œsophagite stade III	33,3	24,4	48,0	28,6	12,5	50,0	NS	NS	NS
Tumeur gastrique	3,9	3,2	6,8	1,9	1,9	2,1	0,1	NS	0,08
Tumeur œsophagienne	0,3	0,4	0,0	0,3	0,0	1,0	NS	NS	NS
Mallory-weiss	1,1	1,4	0,0	1,0	1,4	0,0	NS	NS	-
Autres	3,1	3,2	3,0	3,6	3,8	3,1	NS	NS	NS

Les variables qualitatives sont exprimées par leur fréquence en pourcentage (%). NS : non significatif. Les différences significatives (< 0,05) sont *en italique*. P : comparaison statistique entre les hommes et les femmes

a comparaison entre les hommes et les femmes < 65 ans

b comparaison entre les hommes et les femmes ≥ 65 ans

L'évolution était marquée par un besoin transfusionnel chez 40,1%. Cette fréquence transfusionnelle était plus élevée chez les hommes (42,4 %) que chez les femmes (35,4 %) mais sans différence concernant le nombre de culot globulaire transfusé. Par ailleurs, une récidive hémorragique était notée chez 7,5 % des patients et 5,7 % avaient décédé (Tableau 3).

**Tableau 3:** Caractéristiques évolutives des hémorragies digestives hautes, des hémorragies ulcéreuses et des hémorragies liées à l'hypertension portale (HTP) selon le genre

	Total	Homme	Femme	Signification statistique
**Hémorragies digestives hautes (HDH)**				
Hémostase endoscopique	24,4	21,4	30,8	*1.10* ^*-3*^
Transfusion	40,1	42,4	35,4	*3.10* ^*-2*^
Nombre moyen d'unité transfusée (le cas échéant)	2,6 ± 1,3	2,6 ± 1,3	2,7 ± 1,4	NS
Récidive	7,5	8,3	5,8	0,17
Chirurgie	0,3	0,5	0,0	NS
Décès	5,7	5,5	6,2	NS
**HDH / Ulcère gastroduodénale**				
Hémostase endoscopique	6,3	6,1	7,2	NS
Transfusion	45,8	44,3	51,4	0,18
Nombre moyen d'unité transfusée (le cas échéant)	2,6 ± 1,3	2,6 ± 1,3	2,6 ± 1,3	NS
Récidive	5,4	6,1	2,7	0,15
Chirurgie	0,2	1,0	0,0	NS
Décès	5,2	4,6	7,2	NS
**HDH/ hémorragie liée à l'HTP**				
Hémostase endoscopique	75,3	76,7	73,5	NS
Transfusion	52,1	55,3	47,9	NS
Nombre moyen d'unité transfusée (le cas échéant)	2,5 ± 1,3	2,4 ± 1,0	2,5 ± 1,5	NS
Récidive	13,9	16,0	11,1	NS
Décès	9,7	10,0	9,4	NS

Les variables qualitatives sont exprimées par leur fréquence en pourcentage (%) et les variables quantitatives sont exprimées par leur moyenne ± écart-type. NS : non significatif. Les différences significatives (< 0,05) sont *en italique*.

## Discussion

Parmi les 945 patients ayant une HDH recrutés dans notre formation, la majorité étaient des hommes (67,4%), cette donnée est partagée par plusieurs études occidentales, africaine et méditerranéennes [1–10] ce qui peut être expliqué en partie par la fréquence élevés de l'étiologie ulcéreuse reflétant la prédominance masculine dans la pathologie ulcéreuse gastroduodénale [2, 11–13]. Cette différence dans le sexe ratio des HDH devient moins nette chez les sujets âgés d'au moins 65 ans, puisque les patients de sexes masculin ne représentaient que 58,1 % contre 70,4 % pour les patients plus jeunes (p=5.10^-4^). En effet, les femmes de notre série étaient plus âgées que leurs homologues masculins (47,8 ans versus 51,5 ans) ce qui rejoint les résultats de la littérature [2, 14, 15]. Par ailleurs, les études ayant étudier le changement au fil du temps de l'épidémiologie des HDH laisse suggérer que la proportion des femmes devient de plus en plus importante [16–19], ceci est due à une augmentation de consommation des AINS, chez les femmes âgées notamment, et à une diminution de la prévalence des hémorragies digestives hautes dans la population, plus importantes chez les hommes que les femmes [19–22]. La prise d'AINS ou d'acide acétylsalicylique était identique entre les deux groupes (27,2 % versus 23,4 %). Si l'augmentation du risque, liée à l'âge, de complication gastro-intestinale est bien admise par les différents auteurs, l'influence du sexe sur ce risque est plus controversée [5, 23, 24]; il semblerait que le risque de développer des complications gastro-intestinales induit par les AINS serait plus important chez les hommes à un âge jeune mais devient plus important chez les femmes à un âge avancé [25, 26].

Les femmes, dans notre série, diffèrent des hommes aussi par les signes d'appels puisqu'elles expriment leur hémorragie par une hématémèse, seule ou associée à un méléna, plus fréquemment (85,1 %) que les hommes (79,6 %). Par contre, il n'y avait pas de différence entre les deux groupes concernant les paramètres cliniques ou para-cliniques évaluant l'état hémodynamique des patients et l'importance de l'hémorragie initiale à l'admission (Tableau 1).

Les hommes avaient plus d'ulcère gastroduodénal que les femmes (62 % contre 36,7 %), cette différence devenait plus importante pour les ulcères bulbaires (Tableau 2) mais devenait non significative chez les sujets âgés de plus de 65 ans (51,9 % versus 42,7 %, p = 0,17) et pour l'ulcère gastrique (voir tableau 2). Ces résultats rejoignent ceux de la littérature et pourraient être expliqués d'une part, par un taux de tabagisme plus important chez les hommes, notamment dans notre contexte [27], et une protection contre l'ulcère bulbaire par l'œstrogène chez la population féminine jeune d'autre part [2, 13, 28–31]. Par ailleurs, Cho et al. [32] avaient objectivé que l'âge et le sexe masculin sont deux facteurs indépendants de développement d'une œsophagite, ce qui explique que les patients de sexe masculin âgés de plus de 65 ans avaient une tendance à avoir plus d'œsophagite que les patientes du même âge. Bien que le nombre de femmes ayant une hémorragie liée à l'HTP étaient légèrement inférieur a celui des hommes (H : n = 150 ; F : n = 117), le taux des hémorragies liées à l'HTP étaient plus élevé chez celles-ci (H : 23,5 % ; F : 38,0 %, p = 4.10-6). Ce qui pourrait être expliqué plutôt par les taux bas des hémorragies non liées à l'HTP chez les femmes, notamment la pathologie ulcéreuse. L'étude libyenne d'Elghuel [2] avait trouvé des résultats similaires puisque l'hémorragie par rupture de varice œsophagienne était la première cause chez les patientes de sexe féminin avec un taux plus important que celui des hommes, chez qui l'ulcère hémorragique représentait la première étiologie des HDH. Ce résultat était, par contre, différent de celui des études occidentales qui ont trouvé plutôt une prédominance masculine pour les hémorragies variqueuses [8, 33–35]. Par ailleurs, le taux de gastrite était aussi plus important chez les patientes de sexe féminin par rapport à celui du sexe opposé (Tableau 2) ce qui rejoint les résultats rapportés par d'autres auteurs [2, 36].

Concernant l'évolution, aucune différence n'a été notée concernant les taux de récidive ou de mortalité, globaux ou spécifiques, entre les deux sexes. Quelques études avaient retrouvé le sexe masculin comme facteur de risque de récidive hémorragique [1] et/ou de mortalité [1, 37] des hémorragies digestives hautes mais ces données n'ont pas été confirmées par d'autres études ayant étudié les facteurs pronostiques des HDH [13, 21, 33, 38].

## Conclusion

Notre série a objectivé la présence, chez les patients ayant une hémorragie digestive haute, de différence démographique, clinique et étiologique entre les deux sexes opposés. En effet, les hommes étaient plus fréquents que les femmes mais moins âgés que celles-ci. Si la pathologie ulcéreuse gastroduodénale était la première cause d'HDH chez les patients de sexe masculin, l'hémorragie liée à l'HTP était la première étiologie chez les patientes de sexe féminin. Par ailleurs, l'évolution des malades présentant une HDH était peu influencée par leur genre. Ainsi, la simple connaissance du sexe du patient peut orienter le diagnostic étiologique mais ne peut prévoir le profil évolutif des patients.
